# Fraud Detection in Batches of Sweet Almonds by Portable Near-Infrared Spectral Devices

**DOI:** 10.3390/foods10061221

**Published:** 2021-05-28

**Authors:** Irina Torres, María-Teresa Sánchez, Miguel Vega-Castellote, Dolores Pérez-Marín

**Affiliations:** 1 Department of Bromatology and Food Technology, University of Cordoba, Rabanales Campus, 14071 Córdoba, Spain; g72toroi@uco.es (I.T.); g32vecam@uco.es (M.V.-C.); 2 Department of Animal Production, University of Cordoba, Rabanales Campus, 14071 Córdoba, Spain

**Keywords:** almond batches, authentication, non-targeted fraud detection, non-destructive assessment, *in situ* NIR spectroscopy

## Abstract

One of the key challenges for the almond industry is how to detect the presence of bitter almonds in commercial batches of sweet almonds. The main aim of this research is to assess the potential of near-infrared spectroscopy (NIRS) by means of using portable instruments in the industry to detect batches of sweet almonds which have been adulterated with bitter almonds. To achieve this, sweet almonds and non-sweet almonds (bitter almonds and mixtures of sweet almonds with different percentages (from 5% to 20%) of bitter almonds) were analysed using a new generation of portable spectrophotometers. Three strategies (only bitter almonds, bitter almonds and mixtures, and only mixtures) were used to optimise the construction of the non-sweet almond training set. Models developed using partial least squares-discriminant analysis (PLS-DA) correctly classified 86–100% of samples, depending on the instrument used and the strategy followed for constructing the non-sweet almond training set. These results confirm that NIR spectroscopy provides a reliable, accurate method for detecting the presence of bitter almonds in batches of sweet almonds, with up to 5% adulteration levels (lower levels should be tested in future studies), and that this technology can be readily used at the main steps of the production chain.

## 1. Introduction

Food fraud, which can be defined as an intentional mismatch between a food product’s claims and its actual characteristics, is a growing problem due to the complex nature of food supply chain [1]. Currently, there is an increasing concern about food fraud among authorities, the food industry, and consumers. Food fraud is mainly motivated by economic gain, and could have negative effects on consumers’ health, giving rise to a high level of distrust in food supply chain, as well as a significant economic impact [2,3]. Among the different types of existing fraud, one of the most common is the adulteration, addition, or replacement of an ingredient in order to produce cheaper products.

Recent technological advancements have enabled the emergence of new analytical tools to counter global fraud. Near-infrared spectroscopy (NIRS) is a very powerful technology for food fraud detection, due to its ability to generate a detailed profile or unique fingerprint of each sample analysed, offering a non-destructive, rapid, and high-throughput method of assessment [4]. The use of NIRS in the agri-food sector has benefited from the major technological revolution that has taken place in recent years, which has resulted in new instruments being developed and computer analyses increasing in speed, which in turn has made it possible to combine spectral signals with communications technology in order to obtain reliable data in real time, thus increasing the sampling potential both in the field and industrially [5].

In particular, a new generation of handheld NIRS instruments now enables to analyse products *in situ* [6,7,8] at different stages throughout the food supply chain: on-tree, at reception points in the industry, or in postharvest storage. This allows to analyse the product not only using punctual readings but also by scanning the whole surface, thus obtaining a more representative measurement of the product analysed by incorporating a wider variety of features in the NIRS analysis [9]. However, before NIRS instruments can be used as a routine check to control a product’s authenticity and validity, they need to be evaluated.

In the case of almonds—edible seed drupes which can be categorised as sweet or bitter, depending on their content of the cyanogenic compound amygdalin [10]—the presence of bitter almonds in batches of sweet almonds is an issue in the industry that can cause not only an unpleasant taste and aroma, but problems in commercialization at the national and international levels, and it is therefore of the utmost importance to eradicate bitter almonds from commercialized batches of sweet almonds [11]. Since it is extremely difficult to distinguish bitter almonds from sweet ones visually in adulterated batches, it would be of great interest to the almond sector to be able to use analytical tools with a high throughput which were suitable for continuous and instantaneous discrimination. Thus, the implementation of NIRS sensors in the industrial sorting lines for detecting the adulteration of sweet almond batches with bitter ones could answer this demand.

In this context, it is important to note that there is currently a wide variability in batches of sweet almonds, which makes the discrimination process for bitterness in almonds more difficult. In particular, there are differences in size, shape, weight, and composition, which are mainly dictated by the cultivar to which they belong [12,13]. Therefore, in order to obtain robust discriminant models, training sets need to be set up containing a large number of cultivars of almonds, from various harvesting seasons.

Previous published works have demonstrated the potential of NIRS technology to classify almonds by their bitterness when they are analysed in ground form [14], as individual seeds [11], or by simulating the analysis of batches in the industry [15], but these only discriminate between sweet and bitter almond batches (not between mixtures), and including only one harvesting season. Only one study has demonstrated the possibility of using NIRS technology in almonds to detect cases of non-compliance with the standards established by the industry for batches of sweet almonds, using NIRS fingerprints as a non-targeted control procedure by which to guarantee the integrity of the product when it is received and processed in the industry [16]. Nevertheless, the chemometric strategy applied here does not address the use of discriminant models.

The aim of this research was to investigate the potential of NIRS technology to be implemented at the industrial level to detect batches of sweet almonds adulterated with different percentages of bitter almonds, establishing the lowest limit of bitter almonds that could be detected. Additionally, the influence of the characteristics of the training set samples on the robustness of discriminant models, together with the performance of two portable NIRS sensors, were also evaluated. Moreover, larger and more comprehensive databases, which represent all possible sources of variability, were used to provide a more robust approach to detecting non-compliant batches, which would favour the implementation of NIRS technology as an inspection and authentication tool in the industry.

## 2. Materials and Methods

### 2.1. Sampling

A total of 216 samples—about 750 g per sample—of shelled almonds (*Prunus dulcis* (Mill.)) of different cultivars, harvested during the 2018 (*n* = 145 samples) and 2019 (*n* = 71 samples) seasons, were included in this study (Table 1).

This set comprised 106 samples of the dominant homozygous genotype (*SkSk*, sweet), 30 samples of the heterozygous genotype (*Sksk*, slightly bitter), and 80 samples of the recessive homozygous genotype (*sksk*, bitter). Following the suggestion by Vichi et al. [17], the samples corresponding to the genotypes *SkSk* and *Sksk* were considered to be sweet almonds (N_sweet_ = 136 samples).

First, mixtures (category M) to simulate the adulteration of sweet almond batches with bitter almonds were prepared. To do this, samples were randomly selected from the 136 samples of sweet almonds (category S) and the 80 samples of bitter almonds (category B), with each of the mixtures having a final weight of 500 g. Four types of mixtures were prepared: M_5%_ (95% sweet and 5% bitter almonds), M_10%_ (90% sweet and 10% bitter almonds), M_15%_ (85% sweet and 15% bitter almonds), and M_20%_ (80% sweet and 20% bitter almonds), with a total of 138 mixtures (41 mixtures M_5%_, 39 mixtures M_10%_, 37 mixtures M_15%_, 21 mixtures M_20%_). Of all of these mixtures, 83 were prepared in 2018 (N_M5%_ = 21, N_M10%_ = 21, N_M15%_ = 20, N_M20%_ = 21), and 55 in 2019, focusing only on those mixtures with a lower percentage of bitter almonds (N_M5%_ = 20, N_M10%_ = 18, N_M15%_ = 17). These mixtures were prepared by weighing the corresponding amounts of sweet and bitter almonds using an electronic scale (model PB3002-S, Mettler Toledo, Barcelona, Spain), which were then mixed using a V mixer (Afau, Zaragoza, Spain).

### 2.2. NIRS Instrumentation and Spectrum Acquisition

The spectral acquisition was carried out using two handheld NIRS instruments of different optical designs—a diode array (DA)-based spectrophotometer (Aurora, GraiNit S.r.l., Padova, Italy), and a miniature spectrophotometer based on Linear Variable Filter (LVF) technology, the MicroNIR™ Pro 1700 (VIAVI Solutions, Inc., San Jose, CA, USA).

The DA spectrophotometer is a compact, handheld instrument that carries out spectral acquisition using UCal 4^TM^ software (Unity Scientific LLC, Milford, MA, USA). The instrument has an optical window of about 1256 mm^2^, and works in reflectance mode in the spectral range 950–1650 nm with an interval of 2 nm. In this work, the integration time was set at 6.57 ms, and each spectrum was the mean of 50 scans. Prior to measuring each sample, the instrument was automatically calibrated using an internal reference. For spectral acquisition, each sample of almonds was uniformly distributed on a white plastic tray so that it covered the whole surface. In order to detect as much variability as possible, the spectra were taken in dynamic mode, i.e., moving the sensor along the tray during the measurement, covering its entire area. A total of 4 spectra were taken per sample, which were later averaged to provide a mean spectrum per sample.

The MicroNIR™ Pro 1700 instrument works in reflectance mode in the spectral range 908–1676 nm, with a constant interval of 6.2 nm, and has an optical window of around 227 mm^2^. The sensor integration time was set at 11 ms, and each spectrum was the mean of 200 scans. Spectral acquisition was carried out using VIAVI MicroNIR software Pro version 2.2 (VIAVI Solutions, Inc., San Jose, California, USA). The instrument’s performance was checked every 10 min. To do this, a white reference measurement was obtained using a near-infrared (NIR) reflectance standard (Spectralon^TM^) with 99% diffuse reflectance, while a dark reference was obtained from a fixed point on the floor of the room. The spectral acquisition using the MicroNIR^TM^ Pro 1700 was carried out following the same procedure described above: four spectra per sample were taken in dynamic mode, and these spectra were averaged in order to obtain a mean spectrum per sample.

### 2.3. Study of the Population and Construction of the Training and Validation Sets

The data pre-processing and chemometric treatments were performed using the WinISI II software package version 1.50 (Infrasoft International LLC, Port Matilda, PA, USA) [18] and MATLAB R2018a (The Mathworks, Inc., Natick, MA, USA).

The structure and variability of the population was studied using the CENTER algorithm [19]. This algorithm performs a principal component analysis (PCA), and calculates the global Mahalanobis distance (GH) of each sample to the centre of the population in the new n-dimensional space, which enables the samples to be sorted by their GH distance. The CENTER algorithm was applied using standard normal variate (SNV) and de-trending (DT) as mathematical pre-treatments for scatter correction [20], together with the 1,5,5,1 Norris derivative treatment—where the first digit is the order of the derivative, the second is the gap over which the derivative is calculated, the third is the number of data points in a running average or smoothing, and the fourth is the second smoothing [21]. In this work, this algorithm was individually applied to the sets of sweet and bitter almonds, as well as to the different sets of mixtures (M_5%_, M_10%_, M_15%_ and M_20%_) analysed using both instruments. Those samples which displayed a GH > 4 were studied as potential outliers or anomalous spectra.

To obtain similar sets for the following comparison of results, the same outliers were removed from the datasets analysed with both instruments. Next, a PCA was carried out in order to explore the potential spectral differences between the sweet, bitter, and mixed samples. PCA was performed using the full set of almonds available, and the scores and loadings obtained were studied.

After removing the outliers (one sweet almond sample) and ordering the sets of samples by their spectral distances, the structured selection of the training and validation groups for each set was carried out following the procedure proposed by Shenk and Westerhaus [22]. For the categories of sweet and bitter almonds, 10 samples of each category were selected as test samples in order to validate the model (S_validation_ and B_validation_), while the remaining samples constituted the training sets (S_training_ = 125 samples; B_training_ = 70 samples). In the case of the sets of mixtures, approximately 60% of the samples of each mixture (M_5%_ = 25, M_10%_ = 25, M_15%_ = 25, and M_20%_ = 13) were selected and subsequently merged to form the training set (M_training_ = 88 samples), using the remaining samples (M_5%_ = 16, M_10%_ = 14, M_15%_ = 12, and M_20%_ = 8) to make up the validation set (M_validation_ = 50 samples). The selection was carried out considering all of the sets analysed using the Aurora instrument, and the same samples were selected to make up the training and validation sets for the MicroNIR^TM^ Pro 1700.

### 2.4. Classification Models of Almonds by Bitterness: Influence of the Composition of the Training Sets on the Detection of an Adulterated Product

The classification models of almonds by bitterness were designed using partial least squares-discriminant analysis (PLS-DA). Specifically, the PLS2 algorithm was used, which generates as many discriminant variables as there are classes in the learning group [23]. In order to develop these models, venetian blinds for cross validation (10 splits) were applied, and a maximum of 16 PLS terms was considered. SNV was used as spectral pre-processing for scatter correction [20], and the first and second Savitsky–Golay derivatives treatments were also tested.

For the discrimination analysis, the “sweet” almond category was made up of 100% sweet almonds (samples belonging to category S), while in the case of the “non-sweet” almond category, different strategies were used to construct the training sets (Table 2):

Strategy I: The training set for the “non-sweet” almond category consisted exclusively of 100% bitter almonds (category B).

Strategy II: The “non-sweet” almond category consisted of samples of 100% bitter almonds (category B) and samples belonging to the different mixtures of adulterated sweet almonds containing different percentages of bitter almonds (M_5%_, M_10%_, M_15%_, and M_20%_).

Strategy III.: The “non-sweet” almond category consisted exclusively of samples belonging to the mixtures of sweet almonds adulterated with different percentages of bitter almonds (M_5%_, M_10%_, M_15%_, and M_20%_%).

The performance of the discriminant models was assessed in terms of their sensitivity, specificity, and non-error rate (NER). Due to the fact that the number of samples per category in the training sets was unbalanced, and in order to maximize the success of the models, the optimal threshold value obtained from the receiver operating characteristic (ROC) curves was considered. The discriminant values were established between 0 and 1; for each sample and each category, a value below the threshold ROC value indicated non-membership of the category, and a value over the threshold ROC value indicated category membership.

The validation of the best classification models for each strategy was carried out using the validation sets shown in Table 2.

## 3. Results and Discussion

### 3.1. Characteristics of the NIR Almond Spectra and Study of the Population

**Figure 1:**
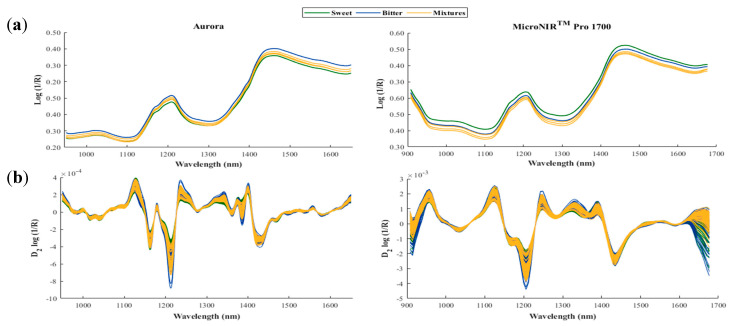
Mean raw (**a**) and second derivative (**b**) spectra of the almond samples analysed using the Aurora and MicroNIR^TM^ Pro 1700 instruments.

The raw mean (Figure 1a) and the pre-processed (Figure 1b) NIR spectra of the three groups of almonds (sweet, bitter, and mixtures) analysed using the two portable NIR instruments displayed very similar features regardless of the group.

For the different groups of samples, the main absorbance bands in the NIR region could be seen at around 1150–1200 nm, linked to the second overtone of C–H stretching, and at around 1400 nm, corresponding to the first overtone of the O–H functional groups [24,25]. Likewise, in the case of the pre-processed spectra (D_2_ log (1/R)) for both instruments (Figure 1b), it can be seen that, in the characteristic absorption zones of the spectra (around 1150–1200 nm and 1350–1440 nm), the DA equipment provides a greater amount of information than the LVF instrument, where some absorption peaks are not detected. This is due to the higher spectral resolution of the DA equipment compared with that of the LVF instrument (measurement interval 6.2 nm vs. 2 nm), which could be an important factor in influencing the success of the classification models subsequently developed.

After carrying out the study of the spectral characteristics, the CENTER algorithm was applied to each spectral group in order to structure the populations. When this algorithm was applied to the sweet and bitter almonds, three samples (two sweet and one bitter) presented a GH value above 4 when the analysis was carried out with the DA instrument. One of the sweet almond samples (GH = 6.09) was removed due to the presence of spectral differences compared with the other samples, while no reasons were found to remove the other two samples. In the case of the groups of mixtures analysed with this instrument, no sample presented GH values above the established limit. As for the samples analysed using the LVF instrument, although no outliers were detected, the same sample as in the diode array database was deleted in order to make up similar training and validation sets for the subsequent comparison between instruments.

**Figure 2:**
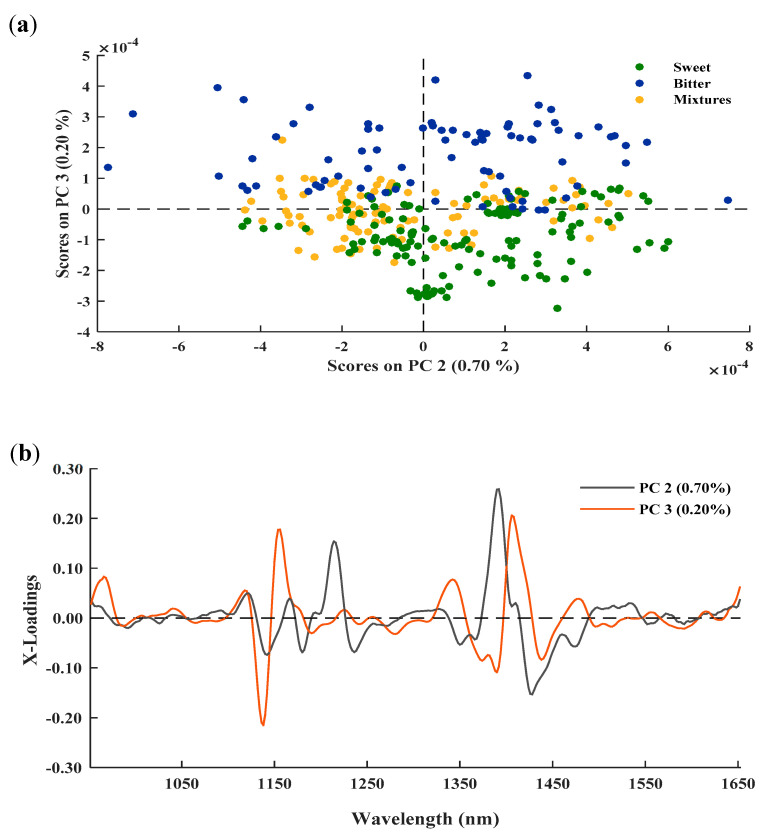
Score plot (**a**) and loading weights (**b**) for the second (PC2) and third (PC3) principal components of the different groups of almonds using the Aurora instrument and the second derivative.

After removing outliers, and prior to the selection of samples to make up the training and validation sets, the structure of the population was assessed by analysing the scores obtained from the PCA of the samples. Although the PCA was developed and studied for both of these instruments, only the DA scores are shown. Figure 2a shows the scores of the second and third principal components (PCs), whereas the PCA loadings for the almonds analysed in the spectral range 950–1650 nm are shown in Figure 2b.

PC2 and PC3, which account for 0.70% and 0.20% of the variance, respectively, are those which permit a clearer distinction between the different groups of almonds. It can be seen that the sweet almonds, as well as the mixtures, tend to present negative PC3 values, while for the bitter almonds, a grouping can be seen where PC3 shows positive values. Likewise, it can be seen that the mixtures are grouped between the two types of almonds—sweet and bitter—although, as expected due to the greater number of sweet almonds they contain, they are closer to the sweet group, and sometimes even overlap with it.

The graphical representation of the X-loadings corresponding to PC2 and PC3 (Figure 2b) were analysed in order to study which bands are the most useful for distinguishing between the different groups of almonds. In the case of PC2, the most prominent peaks are exhibited around 1212 nm, associated with the presence of lipids (C–H second overtone), and at 1390 nm, which could correspond to combination bands of C–H, vibrations and is probably related to fatty acids. It is important to note that in the particular case of almonds, lipids make up around 60% of the total kernel mass, mainly consisting of unsaturated fatty acids, such as the oleic and linoleic acids, which make up 90% of the total fatty acids [26]. In addition, PC3 shows peaks at around 1139–1154 nm, related to the second overtone of the C–H bonds of aromatic compounds, and at the 1424 nm band, which may be due to the first overtone of the O–H functional groups [24,25].

### 3.2. Development of Classification Models to Detect Adulterated Batches of Sweet Almonds

#### 3.2.1. Strategy I

Table 3 shows the results of the best classification models obtained following Strategy I to distinguish between the sweet and bitter almonds analysed using the Aurora and MicroNIR^TM^ Pro 1700 instruments. This was the first derivative mathematical treatment, which yielded the best classification statistics in cross-validation for both instruments.

For the “sweet” category, 100% of the samples were correctly classified using the DA instrument, and 99% (124/125) with the LVF spectrophotometer, whereas for the “non-sweet” category (in this case made up of 100% bitter almonds), 100% (70/70) were correctly classified for the models developed with both instruments. The wrongly classified sample of the “sweet” category with the LVF instrument was a sample belonging to the *Largueta* cultivar (genotype *SkSk*), which is characterized by large, elongated, and flat kernels, resulting in an amygdalin content of 56.69 mg kg^−1^, higher than the usual mean content for this cultivar (40.62 mg kg^−^^1^).

Next, the actual situation in the industry where this technique would be applied to detect possible batches of sweet almonds adulterated with different percentages of bitter almonds was considered. Therefore, the external validation of the best models obtained for the two instruments tested, using a set including a total of 158 samples (10 samples of the “sweet” category, and 148 belonging to the “non-sweet” category, including 10 bitter samples and 138 mixtures) was carried out.

Table 3 shows that all of the samples of 100% sweet and bitter almonds used as controls were correctly identified using both instruments. When it came to predicting the adulterated mixtures, the results were poor: with the DA instrument, the total percentage of mixtures identified as belonging to the “non-sweet” category was only 38% (52/138), while with the LVF instrument, just 20% (27/138) of the mixtures were correctly classified.

#### 3.2.2. Strategies II and III

Given that the results obtained with Strategy I for the construction of discriminant models, using only 100% sweet or 100% bitter samples in the training sets, did not provide adequate results for predicting the mixtures (adulterations of batches of sweet almonds with a 5–20% content of bitter almonds), other strategies for setting up a training set that would allow to increase the model’s predictive capacity when applied to mixtures or adulterated batches of sweet almonds with different percentages of bitter almonds (Strategies II and III) were evaluated.

The results obtained in cross-validation for the best classification models for Strategies II and III, and the two instruments, are shown in Table 4. These results are substantially better than those obtained with Strategy I.

Thus, for the DA instrument, the percentages of samples correctly classified with Strategy II were 97% and 99% for the categories defined as “sweet” and “non-sweet”, respectively, while with Strategy III, 96% and 97% of the samples of the two abovementioned categories, respectively, were correctly classified. For the LVF instrument, with the two strategies tested, the classification capacity obtained was lower than that of the DA instrument, with success rates of 87% for the “sweet” category (both strategies) and 92% and 90% for the “non-sweet” category (Strategies II and III, respectively).

For the two instruments evaluated, as can be seen, although the success of the model for the “sweet” category is similar in the two strategies studied, the classification capacity for the “non-sweet” category is slightly higher with Strategy II than with Strategy III, which may be due to the effect the increased number of samples included in each of the classes has on the development of the model and, therefore, its degree of representativeness [23,27].

The results for three of the samples of the “sweet” category wrongly classified by the DA instrument—two of the *Largueta* cultivar (*SkSk*), and one of the *Garrigues* cultivar (*Sksk*)—were the same with the two strategies used. After a more detailed study, we were able to determine that the sample belonging to the *Largueta* cultivar was the sweet sample that had been misclassified with the LVF instrument in Strategy I and that, as previously mentioned, presented a content of amygdalin (56.69 mg kg^−1^) higher than the mean content of amygdalin (40.62 mg kg^−1^) for the samples of this variety (Table 1). Similarly, it should be noted that these three samples of the “sweet” category misclassified with the DA instrument using both strategies were also misclassified with the LVF instrument, regardless of the strategy followed. In addition, with the other wrongly classified samples of the “sweet” category analysed with the LVF equipment, regardless of the strategy followed, a high percentage of these samples (75% and 63% for Strategies II and III, respectively) belonged to cultivars of which a limited number of samples were available and, therefore, had a low representation in the training set. In the particular case of the cultivar *Avellanera*, all of the samples of this cultivar included in the training set (*n* = 4) were wrongly classified. Although the amygdalin content of this cultivar was low, the greater difficulty in classifying this cultivar could be due to the fact that it presented different physical characteristics from the rest of the samples analysed, with a very small, rounded grain size. This seems to confirm the importance of having large databases to increase the representativeness of the training sets in order to accurately predict unknown samples in the future [28].

Regarding cross-validation in the “non-sweet” category, and following Strategy II, all of the 100% bitter samples included in the training set were correctly classified with the two instruments used, while in the case of the mixtures, greater success of the model was achieved with the DA equipment than with the LVF instrument, correctly identifying 98% (86/88) versus 86% (76/88) of the mixtures, respectively. Of the two samples wrongly classified with the DA instrument (one from M_5%_ and one from M_15%_), one was prepared with a sample of the *Belona* cultivar and the other used a cultivar of bitter almond which had a low representation in the training set. In addition, a study of the samples wrongly classified by the LVF instrument revealed that of the 12 misclassified samples (3 M_5%_, 1 M_10%_, 5 M_15%_, and 3 M_20%_), 6 were prepared with the cultivar *Belona*. This coincides with the results obtained in previous research by Vega-Castellote et al. [16], who found greater difficulty in identifying mixtures prepared using *Belona* as a non-sweet almond, possibly due to its shape.

In the case of the models developed using Strategy III to construct the training sets, both instruments identified all of the samples of the 100% bitter category in cross-validation, although in the case of the mixtures, 97% (85/88) were correctly classified by the DA instrument compared to 90% (79/88) with the LVF. As with Strategy II, two of the samples wrongly classified using the DA equipment and four using the LVF were prepared with the *Belona* cultivar, thus confirming the complexity of identifying batches of samples in this cultivar, at least when it does not make up the majority of the sample.

Although the results obtained with the two instruments used are highly promising for the detection of fraudulent batches of almonds, the DA instrument achieved greater success with classification (>96%) than the LVF instrument (>87%) for both strategies. These differences between the two instruments could be due to the larger window size and resolution of the DA spectrophotometer. In view of the results obtained, itcan confirm that, in the case of highly heterogeneous products, such as mixtures, it is essential to analyse a larger surface area of the product in order to detect the maximum amount of variability. This factor is decisive in the success of the model with a view to classifying samples in the future, and it is therefore essential to carry out an exhaustive sampling procedure in order to obtain more complete, precise information on the whole product analysed [29].

### 3.3. External Validation: Strategies II and III

The models developed with the two NIRS instruments were externally validated, although only the results obtained with the DA instrument are shown here, due to its greater classification capacity. The results obtained in the external validation of the models developed with the DA instrument following Strategies II and III are shown in Table 5.

**Figure 3:**
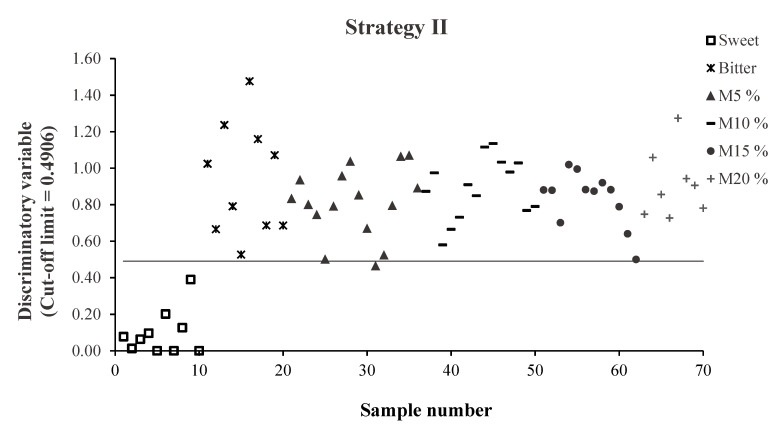

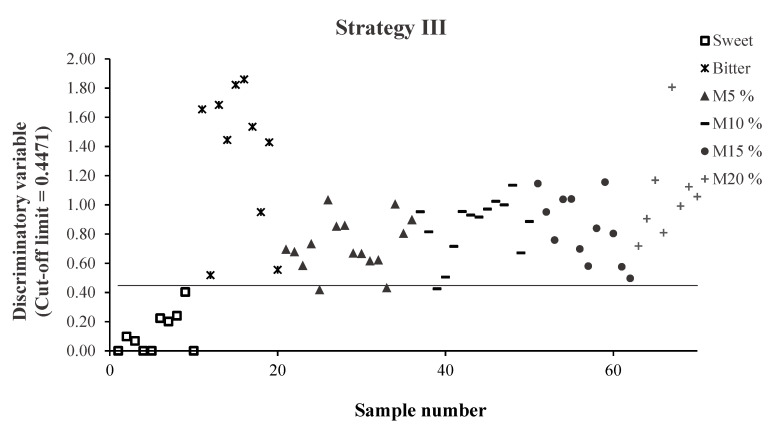
Values of the discriminatory variable for each sample of the validation set in the “non-sweet” category, obtained for Strategies II and III using the Aurora instrument.

Furthermore, discriminatory variable values obtained for each sample in the validation set following Strategies II and III are shown in Figure 3. Since the objective of the qualitative analysis was to detect those batches that contained bitter almonds, Figure 3 shows the discrimination values assigned to each sample in the “non-sweet” category, displaying values close to 0 for the samples belonging to the “sweet” category and values approaching 1 for the “non-sweet” category. Thus, values above the cut-off limit established by the ROC curve for each strategy indicate that the sample belongs to the “non-sweet” category, whereas a value below the limit indicates non-membership of that category, i.e., belonging to the “sweet” category.

The comparison of the results obtained following Strategies II and III (Table 5) showed that Strategy II allowed to detect a higher number of adulterated samples. For the two strategies used, 100% of the samples of sweet almonds and bitter almonds were correctly classified. In addition, 98% (49/50) of the mixtures from the external validation group were detected and identified as “non-sweet” following Strategy II (all of the mixtures except one adulterated with 5% bitter almonds), while with Strategy III, this percentage was slightly lower, at 94% (47/50). In this case, all of the mixtures were classified correctly, except for one adulterated with 10% and two with 5% bitter almonds. It is worth mentioning that, in the case of Strategy II (Figure 3), the 5% mixture that was not identified as “non-sweet” showed a discriminatory value (0.4656) close to the established limit of 0.4906.

As regards the misclassified mixtures (two of 5% and one of 10%) using Strategy III (Figure 3), it is important to note that, in two of them (one M_5%_ and one M_10%_) corresponding to the 2019 season, bitter almonds with different characteristics to the rest of the samples of this category were used. These almonds were taken from an experimental field, and only one sample per cultivar was available, so they had a low representation within the training group. Although these two samples were correctly classified using Strategy II, this fact illustrates the importance of having broad training groups which include samples of 100% bitter almonds. This would allow better detection of batches of adulterated almonds, mainly in those cases in which less widespread—and thus, less represented—cultivars are included.

Figure 3 shows, for Strategy III, a stratification of the “non-sweet” category samples based on their percentage of bitter almonds. It is particularly interesting to note that a high percentage (70%) of the 100% bitter almonds samples (category B) present a discriminatory variable value well above 1, and above the value obtained for the mixtures, while in the case of the samples of sweet almonds, with the exception of one sample, all of the values were well below the limit. This sample, despite being correctly classified (discriminatory value = 0.4031), was a sample of the cultivar *Avellanera*, which, as mentioned above, could be a cultivar with uncommon morphological characteristics, which might affect its classification.

## 4. Conclusions

The results obtained enable to propose NIRS technology, using two portable instruments based on DA and LVF technologies, to be used as a non-destructive assessment method to detect batches of sweet almonds adulterated with bitter almonds, thus helping to stop bitter almonds from being included in the processed product, which detracts from their commercial value. Based on the results obtained, Strategy II—which includes samples of 100% bitter almonds, samples of 100% sweet almonds, and samples of mixtures in the training set—was established to be the best strategy for detecting batches of almonds containing different percentages of bitter almonds prior to their incorporation in the processing line. As can be seen with the mixtures tested in this study, the detection limit of bitter almonds could be set at 5%. In future works, in order to reduce this limit, it would be of particular interest to use a larger sample group including mixtures with percentages of adulterated almonds below 5%.

## Figures and Tables

**Table 1:** Genotype and amygdalin content (mg kg^−1^) of the different cultivars analysed.

Genotype	Cultivar	Range	Mean	Standard Deviation	Coefficient of Variation (%)
*SkSk*	*Antoñeta*	194.80–349.40	284.72	44.12	15.50
	*Avellanera*	0.00–59.20	16.74	21.72	129.75
	*Belona*	11.64–150.84	62.45	36.61	58.62
	*Blanquilla*	25.30–229.60	100.96	75.20	74.48
	*Comuna*	37.60–230.90	115.00	70.81	61.57
	*Ferragnes*	0.00–18.80	10.94	6.86	62.71
	*Largueta*	0.00–71.90	40.62	25.13	61.87
	*Laureanne*	5.45–131.02	62.53	35.89	57.40
	*Marcona*	72.90–138.00	113.30	21.68	19.14
	*Ramillete*	0.00–56.60	29.28	23.99	81.93
	*Soleta*	77.05–165.95	112.37	25.53	22.72
	*Vairo*	26.88–125.32	62.59	27.20	43.46
*Sksk*	*Garrigues*	82.20–137.90	104.40	24.10	23.08
	*Guara*	0.00–551.92	224.06	148.62	66.33
*sksk*	-	215.03–80,980.13	34,508.14	30,173.61	87.44

**Table 2:** Characterization of training and validation sets for the different strategies tested for the construction of the training sets.

	Strategy I		Strategy II		Strategy III	
	“Sweet” Almond Class	“Non-Sweet” Almond Class	“Sweet” Almond Class	“Non-Sweet” Almond Class	“Sweet” Almond Class	“Non-Sweet” Almond Class
**Training set**	100% sweet almonds (*n* = 125 samples)	100% bitter almonds (*n* = 70 samples)	100% sweet almonds (*n* = 125 samples)	100% bitter almonds (*n* = 70 samples) + M_5%_ (*n* = 25 samples) + M_10%_ (*n* = 25 samples) + M_15%_ (*n* = 25 samples) + M_20%_ (*n* = 13 samples)	100% sweet almonds (*n* = 125 samples)	M_5%_ (*n* = 25 samples) + M_10%_ (*n* = 25 samples) + M_15%_ (*n* = 25 samples) + M_20%_ (*n* = 13 samples)
**Validation set**	100% sweet almonds (*n* = 10 samples)	100% bitter almonds (*n* = 10 samples) + M_5%_ (*n* = 41 samples) + M_10%_ (*n* = 39 samples) + M_15%_ (*n* = 37 samples) + M_20%_ (*n* = 21 samples)	100% sweet almonds (*n* = 10 samples)	100% bitter almonds (*n* = 10 samples) + M_5%_ (*n* = 16 samples) + M_10%_ (*n* = 14 samples) + M_15%_ (*n* = 12 samples) + M_20%_ (*n* = 8 samples)	100% sweet almonds (*n* = 10 samples)	100% bitter almonds (*n* = 10 samples) + M_5%_ (*n* = 16 samples) + M_10%_ (*n* = 14 samples) + M_15%_ (*n* = 12 samples) + M_20%_ (*n* = 8 samples)

**Table 3:** Classification of intact almonds by bitterness using the Aurora and MicroNIR^TM^ Pro 1700 instruments. Strategy I.

	Instrument									
	Aurora					MicroNIR^TM^ Pro 1700				
			**Predicted Class**					**Predicted Class**		
	**Actual Class**		Sweet	Non-Sweet	Samples Correctly Classified	Actual Class		Sweet	Non-Sweet	Samples Correctly Classified
**Cross-validation**	Sweet		125	0	100.00%	Sweet		124	1	99.20%
	Non-sweet		0	70	100.00%	Non-sweet		0	70	100.00%
			**Sensitivity = 1**	**Specificity = 1**	**NER = 100%**			**Sensitivity = 0.99**	**Specificity = 1**	**NER = 99.49%**
**External validation**			**Predicted Class**					**Predicted Class**		
	**Actual Class**		Sweet	Non-Sweet	Samples Correctly Classified	Actual Class		Sweet	Non-Sweet	Samples Correctly Classified
	Sweet		10	0	100.00%	Sweet		10	0	100.00%
	Non-sweet	Bitter	0	10	100.00%	Non-sweet	Bitter	0	10	100.00%
		M_5%_	31	10	24.39%		M_5%_	35	6	14.63%
		M_10%_	24	15	38.46%		M_10%_	30	9	23.08%
		M_15%_	24	13	35.14%		M_15%_	28	9	24.32%
		M_20%_	7	14	66.67%		M_20%_	18	3	14.29%
			**Sensitivity = 1**	**Specificity = 0.42**	**NER = 45.57%**			**Sensitivity = 1**	**Specificity = 0.25**	**NER = 29.75%**

**Table 4:** Discriminant models for classifying almond batches by bitterness, analysed with the Aurora and MicroNIR^TM^ Pro 1700 instruments. Strategies II and III. Cross-validation.

Instrument								
	Aurora				MicroNIR^TM^ Pro 1700			
		Predicted Class				Predicted Class		
	**Actual Class**	Sweet	Non-Sweet	Samples Correctly Classified	Actual Class	Sweet	Non-Sweet	Samples Correctly Classified
**Strategy II**	Sweet	121	4	96.80%	Sweet	109	16	87.20%
	Non-sweet	2	156	98.73%	Non-sweet	12	146	92.41%
		**Sensitivity = 0.97**	**Specificity = 0.99**	**NER = 97.88%**		**Sensitivity = 0.87**	**Specificity = 0.92**	**NER = 90.11%**
**Strategy III**	Sweet	120	5	96.00%	Sweet	109	16	87.20%
	Non-sweet	3	85	96.59%	Non-sweet	9	79	89.77%
		**Sensitivity = 0.96**	**Specificity = 0.97**	**NER = 96.24%**		**Sensitivity = 0.87**	**Specificity = 0.88**	**NER = 88.26%**

**Table 5:** External validation of the models developed to detect adulterated almond samples using the Aurora instrument, following Strategies II and III.

Strategy II	Actual Category		Classified as		Correctly Classified
			Sweet	Non-Sweet	
	Sweet		10	0	100.00%
	Non-sweet	Bitter (M_100%_)	0	10	100.00%
		M_5%_	1	15	93.75%
		M_10%_	0	14	100.00%
		M_15%_	0	12	100.00%
		M_20%_	0	8	100.00%
			**Sensitivity = 1**	**Specificity = 0.98**	**NER = 98.57%**
**Strategy III**	**Actual Category**		**Classified as**		**Correctly Classified**
			**Sweet**	**Non-Sweet**	
	Sweet		10	0	100.00%
	Non-sweet	Bitter (M_100%_)	0	10	100.00%
		M_5%_	2	14	87.50%
		M_10%_	1	13	92.86%
		M_15%_	0	12	100.00%
		M_20%_	0	8	100.00%
			**Sensitivity = 1**	**Specificity = 0.95**	**NER = 95.71%**
